# Editorial: Non-Genetic Heterogeneity in Development and Disease

**DOI:** 10.3389/fgene.2021.731814

**Published:** 2021-08-09

**Authors:** Jean-Pascal Capp, Mohit Kumar Jolly, Ankur Sharma

**Affiliations:** ^1^Toulouse Biotechnology Institute, INSA/University of Toulouse, CNRS, INRAE, Toulouse, France; ^2^Centre for BioSystems Science and Engineering, Indian Institute of Science, Bangalore, India; ^3^QEII Medical Centre and Centre for Medical Research, Harry Perkins Institute of Medical Research, The University of Western Australia, Perth, WA, Australia; ^4^Curtin Medical School, Curtin Health Innovation Research Institute, Curtin University, Perth, WA, Australia

**Keywords:** cancer, inflammation, epigenetics, plasticity, transcription

Genetically identical populations of mammalian cells can demonstrate inherent heterogeneity in gene expression and consequent functional behavior. This non-genetic heterogeneity can emerge due to stochasticity in gene expression, plasticity emerging due to gene regulatory networks, and the epigenetic state of cells. The non-genetic heterogeneity of cells can determine cell fate decision and differential response and adaptation to varying environmental conditions. Non-genetic heterogeneity of stromal and tissue resident stem cells are also known to play an important role in development, cancer, and infectious diseases. Thus, non-genetic heterogeneity is emerging as a major player in mediating resistance to existing therapies. In this Research Topic, we aimed to assemble a collection of manuscripts that address the following important questions: What is the major source of non-genetic heterogeneity, and what is its role in homeostasis and pathobiology? Why and how does non-genetic resistance occur?

After exploring the literature revealing the stochastic nature of cell differentiation and the role of stochastic gene expression (the so-called “gene expression noise”) in this process, Capp and Laforge propose to consider an alternative model of development named ontophylogenesis where the generation of a differentiated state is considered as a constrained random process. The chance-selection principle governing cell differentiation would be based on the randomness of biochemical reactions at lower scales on which the multiscale constraints produced by the dynamical organization of the biological system retroact, thus driving the system toward a stabilized state of equilibrium. Mitchell highlights the experimental and computational systems biology studies that have been instrumental in decoding how B-cells achieve distinct fates and the implications of various mutations. The article focuses on mechanisms leading to cell-to-cell variability in B-cell terminal differentiation, and consequences on population heterogeneity in terms of decision-making timings and population distribution proportions. Next, Sha et al. demonstrate using single-cell transcriptomic data on epithelial-mesenchymal transition (EMT) that intermediate cell-states along the EMT spectrum can play a crucial role in TGFb-induced EMT. Analyzing the trajectory of cell-state transitions induced by various growth factors, they highlight how intermediate states can be instrumental for cell-cell communication, highlighting a role of non-cell-autonomous factors in decision-making. Tonn et al. discuss the “metabolic” phenotypic heterogeneity at single cell resolution. They propose a mixture model for systematic prediction of the impact of biochemical parameters on the metabolite distribution at single cell level. This study opens the avenue for uncharted territory of single cell metabolic heterogeneity.

Gessain et al. discuss the non-genetic heterogeneity in the immune system particularly in macrophages. They specifically discuss the heterogeneity of macrophages in human diseases in the context of infection, inflammation, metabolism, aging and cancer. Finally they summarize how non-genetic heterogeneity in macrophages may impact the pathophysiology in humans and its implication in therapeutic targeting. Specifically on cancer, Guinn et al. propose in a stimulating article that the role of gene expression noise in metastasis should be investigated by two complementary approaches. On the one hand, the authors discuss the monitoring and cataloging of naturally occurring gene expression variability to establish associations with cancer progression and metastasis, and suggest that three different types of noise-modulated threshold crossing (multistability, hypersensitivity, and irreversibility) should be more particularly studied in the context. On the other hand, they propose to experimentally modulate protein noise independently of the mean through synthetic biological gene circuits to confirm the role of non-genetic heterogeneity in disease development, stress survival, and metastasis.

From a therapeutic viewpoint, Biswas discusses the phenotypic heterogeneity in treatment response, specifically the heterogeneity in cellular response and downstream signaling and its impact on treatment response. Interestingly, he discusses a similar mechanism of action during heterogeneous cellular responses. Farquhar et al. discuss how a combination of computational and experimental approaches helps decoding the design principles of fractional killing and non-genetic heterogeneity implicated in antimicrobial resistance (AMR). They also expound the implications of these ideas in cancer drug resistance and underscore the importance of synthetic biology attempts based on the design principles of regulatory networks, which can help discover effective strategies against AMR.

Deshmukh and Saini adopt a broader perspective by considering the evolutionary implications of phenotypic heterogeneity at all levels of life, from viruses to mammals. On the one side, the authors particularly emphasize the role of non-genetic variability during organismal development (with *Caenorhabditis elegans* as an example) or within a specialized organ system (specifically spermatogenesis). On the other side, its potential initiating and promoting role in the onset of cancer is largely discussed, with detailed examples over apoptosis, signaling, metabolism, as well as drug resistance. Finally, moving beyond investigating phenotypic plasticity and heterogeneity, Clairambault takes the focus on cancer progression from an evolutionary perspective, presenting a breakdown of multicellularity as one of the hallmarks of cancer. The article asks poignant questions about the connection between emergence of multicellularity and that of cancer, and argues that investigating how multicellularity originated can have important insights into understanding how it breaks down during multiple stages of cancer progression. This series of stimulating articles highlight that non-genetic heterogeneity should be considered as a central component in development and disease, and reveal that innovative theories and experiments in modern biology can be elaborated and conducted by considering non-genetic heterogeneity as a driving force in physiological and pathological systems.

## Author Contributions

J-PC, MJ, and AS conceived, wrote, and edited the final version of this editorial. All authors contributed to the article and approved the submitted version.

## Conflict of Interest

The authors declare that the research was conducted in the absence of any commercial or financial relationships that could be construed as a potential conflict of interest.

## Publisher's Note

All claims expressed in this article are solely those of the authors and do not necessarily represent those of their affiliated organizations, or those of the publisher, the editors and the reviewers. Any product that may be evaluated in this article, or claim that may be made by its manufacturer, is not guaranteed or endorsed by the publisher.

